# Mechanistic insight into the functional transition of the enzyme guanylate kinase induced by a single mutation

**DOI:** 10.1038/srep08405

**Published:** 2015-02-12

**Authors:** Yuebin Zhang, Huiyan Niu, Yan Li, Huiying Chu, Hujun Shen, Dinglin Zhang, Guohui Li

**Affiliations:** 1 State Key Laboratory of Molecular Reaction Dynamics, Dalian Institute of Chemical Physics, Chinese Academy of Sciences, 457 Zhongshan Rd, Dalian 116023, P.R. China; 2 Department of Geriatrics, Shengjing Hospital, China Medical University, 36 Sanhao Street, Heping, Shenyang 110004, P. R. China

## Abstract

Dramatic functional changes of enzyme usually require scores of alterations in amino acid sequence. However, in the case of guanylate kinase (GK), the functional novelty is induced by a single (S→P) mutation, leading to the functional transition of the enzyme from a phosphoryl transfer kinase into a phosphorprotein interaction domain. Here, by using molecular dynamic (MD) and metadynamics simulations, we provide a comprehensive description of the conformational transitions of the enzyme after mutating serine to proline. Our results suggest that the serine plays a crucial role in maintaining the closed conformation of wild-type GK and the GMP recognition. On the contrary, the S→P mutant exhibits a stable open conformation and loses the ability of ligand binding, which explains its functional transition from the GK enzyme to the GK domain. Furthermore, the free energy profiles (FEPs) obtained by metadymanics clearly demonstrate that the open-closed conformational transition in WT GK is positive correlated with the process of GMP binding, indicating the GMP-induced closing motion of GK enzyme, which is not observed in the mutant. In addition, the FEPs show that the S→P mutation can also leads to the mis-recognition of GMP, explaining the vanishing of catalytic activity of the mutant.

Exhibiting functional diversity of proteins usually requires a large number of sequence alterations through multiple evolutionary steps^1^,^2^. However, in the case of the guanylate kinase (GK) enzyme^3^,^4^,^5^, functional novelty is observed in a single (S→P) mutation, leading to the conversion of the protein from a phosphoryl transfer kinase into a phosphorprotein interaction domain^6^,^7^.

The GK enzyme is a broadly distributed nucleotide kinase and essential for cellular GMP recycling and nucleotide equilibration^5^. The protein is involved in a crucial intermediate step in RNA or DNA synthesis by catalyzing the phosphoryl group transfer from ATP to GMP^3^. According to the available X-ray crystallography structures of GK enzyme^6^,^7^,^8^,^9^, it can be structurally divided into three featured domains: the N-terminal GMP-binding domain (GBD), the C-terminal ATP -binding LID domain and the innermost CORE domain. In the apo form, the protein resembles a U-shape.Upon the substrate GMP binding to the binding site between the GBD and LID domain, large conformational changes would be induced, leading to the formation of a fully closed state, which is analogous to the closing motion of the fingers and the thumb^7^,^10^,^11^,^12^.

Recently, Johnston et.al^7^ demonstrated that a single mutation (S35P) of GK enzyme would fundamentally alter the protein function and switch it from a phosphoryl transfer kinase into a phosphoprotein interaction domain to regulate spindle orientation. In addition, the phylogenetic analysis suggested that the GK enzyme could evolve into a protein interaction domain, namely the guanylate kinase (GK) domain, with significantly high structural similarity and sequence identity^13^,^14^. The GK domain is one of the core modules of the scaffold protein Membrane Associated Guanylate Kinase (MAGUK), which is responsible for modulating cell-cell communication, spindle orientation, and cellular signal transduction^15^,^16^,^17^,^18^,^19^,^20^,^21^,^22^. Functionally, the GK domain is able to interact with a variety of phospho-peptide ligands with high affinity but its binding ability to GMP is surprisingly low^18^. Previously, Olsen et.al^10^ reported that mutating the Ser35 of GK enzyme to Pro, a conversed proline residue at this position among all GK domains, drastically impairs the GMP binding affinity and significantly reduces the guanylate kinase activity. By comparing the difference between the crystal structures of GK enzyme^6^,^7^,^8^,^9^ and GK domain^22^, it can be observed that eight residues from the GK enzyme directly contact with the ligand and three residues (Ser35,Glu70 and Asp101) more closely coordinate to the guanine ring of GMP, while the other five residues remain invariant from the GK enzyme to the GK domain.

Thus, the experimental evidences mentioned above clearly highlight the critical functional role of Ser35 in the GK enzyme. In this work, in order to elucidate the underlying mechanism of its functional transition after introducing the proline at position 35 of GK enzyme, we carried out molecular dynamic (MD) simulations to investigate their dynamic behaviors. In consistence with experimental observations, our simulations demonstrate that the S→P mutation fundamentally changes the dynamic motion of the protein, leading to the dynamic behavior of the mutant more similar to the GK domain.

We further investigated the relationship between the conformational transitions and GMP binding in both WT and S35P GK by employing the bias-exchange metadynamics^23^. Our calculated free energy profiles (FEPs) suggest that the closed-open transition of WT GK is positive correlated with the process of GMP binding, indicating the GMP-induced closing motion of GK. In contrast, the S → P mutation raises a barrier for the closing motion and leads to the protein energetically favoring the open conformation even with the presence of GMP. Moreover, the FEPs further demonstrate that mutating Ser35 to Pro also results in the mis-binding of GMP in the binding site, explaining the vanishing of catalytic activity of the mutant.

## Methods

### System Preparations

**Figure 1:**
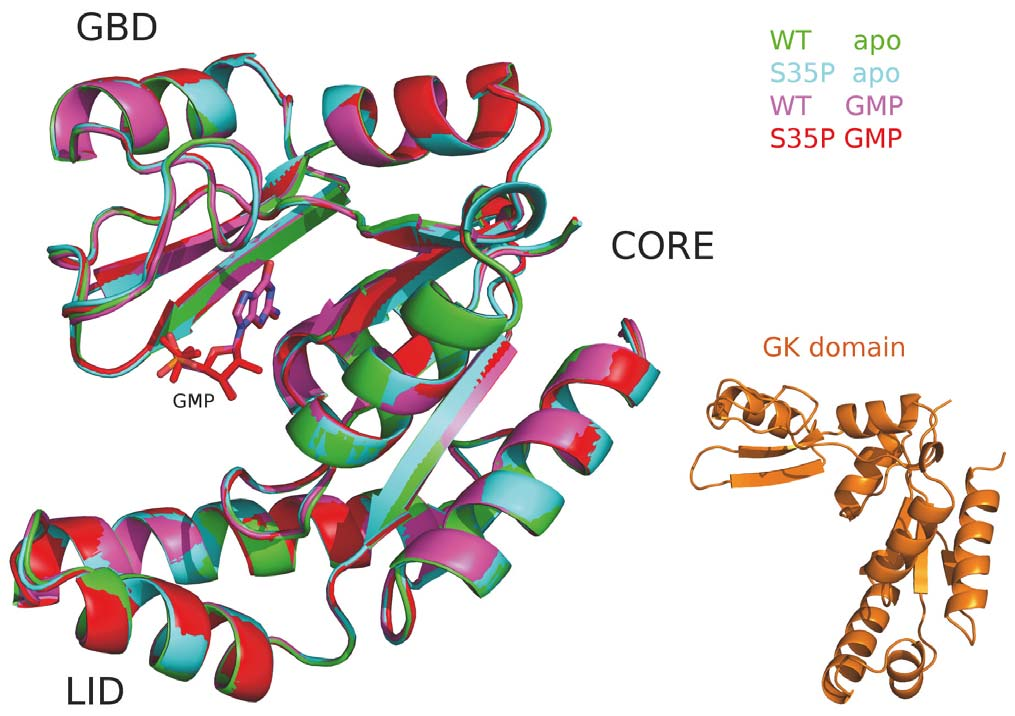
The superimpose of initial conformations of the GK enzymes (green:apoWT; cyan:apo S35P mutant; magenta:GMP-bound WT;red:WT-bound S35P mutant) and the GK domain (orange).

The structure of the GMP-binding form of WT GK enzyme (PDBid:1ex7;186 amino acids)^8^ was used as the initial conformation for theoretical modeling. The apo form of WT GK enzyme was prepared by removing the GMP ligand from its GMP-binding form. The Mutator Plugin in the VMD program^24^ was utilized to mutate Ser to Pro at position 35 of GK enzyme for both the apo- and the GMP-binding forms. Accordingly, as shown in Figure 1, all of the starting structures have the identical backbone conformations except for the GK domain which is extracted from the MAGUK scaffold protein (PDBid:3uat)^22^.

### Standard MD Simulations

All MD simulations were performed using the Gromacs^25^,^26^ MD simulation engine with amber03 force field^27^. The MD protocol for all systems is given as follow. For each system, the GK enzyme was centered into a ~80 × 80 × 80 Å cube, and then was dissolved in ~18000 TIP3 waters. Then, 0.1 M NaCl ions were added to neutralize the net charge of the whole system. The steepest descent algorithm was employed to minimize the whole system before it was gradually heated to 300 K with a increment of 20 K within 50000 steps. The leap-frog integrator was used to produce a 300 ns unbiased MD trajectory for each system with an integration time-step of 2 fs under NPT condition. The Parrinello-Rahman barostat^28^ was used to control the pressure at 1 bar with a coupling constant of 2ps. The modified Berendsen (V-rescale) thermostat^29^ was employed to control the temperature of the systems at 300 K with a time constant of 1 ps. The Particle Mesh Ewald method^30^ was used to compute the electrostatic interactions with a real-space cut-off distance of 1 nm. The same cutoff value was chosen for treating the van der Waals interactions. The SETTLE algorithm^31^ was used to constrain water molecules and all non-water bonds were constrained using the LINCS algorithm^32^.

### Free Energy Profile

Metadynamics^33^,^34^,^35^,^36^,^37^,^38^,^39^,^40^ is a recently developed enhanced sampling technique used to explore the free energy profile (FEP) of a system along coarse-grained reaction coordinates, usually a few collective variables (CVs). In this work, according to the analysis of the RMSF results for essential dynamic, a collective variable (CV1) was defined as the distance between the Cα atoms of Gly43 and Thr137 and another collective variable (CV2) was considered as the distance between the carbonyl oxygen of GMP and the side chain of residue 35 in WT GK (the hydroxyl group of Ser35) and in S35P GK (the side chain of Pro35), respectively. The method was depicted as “filling the free energy wells with computational sand” which exert a positive user-defined Gaussian potential intermittently to the real-energy landscape of the system in a chronological order. Thus during the evolution of the simulation, more and more Gaussians were summed up and not updated afterwards until the system explored the full energy landscape. In the long time limit, the bias potential converges to minus the free energy. 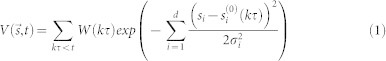

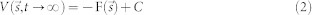


In this work, we carried out the bias-exchange (BE) matadynamics^34^,^35^ in Gromacs/PLUMED^41^,^42^ to obtain the 2D free energy profiles. In BE matadynamics, several replicas run in parallel and the configurations between pairs of replicas are allowed to swap at a fixed time interval. We chose the CV1 and CV2 in replica 1 and allow the exchange between configurations with the unbiased system (replica 2) using an exchange rate of 120 ps. On average, the exchange acceptance ratio was ~27% between the two replicas. The MD protocol for the BE matadynamics were applied similarly as the standard MD simulations mentioned above. The Gaussian widths σ were set to 0.1 for CV1 and 0.01for CV2 with the height of W = 0.1 kJ/mol added by every 20ps. Well-tempered metadynamics^43^ were also employed to avoid overfilling with BIASFACTOR = 10 at TEMP = 300 K. The convergence of FEP in each basin is estimated as a function of simulation time.

## Results and Discussion

Computational investigations of protein conformational changes and ligand binding events have become indispensable tools to provide the atomic description of protein functions for the experimental observations^44^,^45^,^46^,^47^. However, sampling large-scale conformational space of the protein dynamic and acquiring the energetic profile of a ligand binding process in standard molecular dynamic (MD) simulations are still challenging tasks. In this work, by employing standard MD simulations and metadynamics, we provide a detailed description of the conformational transitions and ligand binding process for the WT GK enzyme and the S35P mutant to illustrate the mechanism of its functional transitions. To compare the dynamical motions between the WT and the S35P GK, we first conduct standard MD simulations for both of their ligand-free and ligand-bound forms. An additional simulation of the GK domain is also performed as a comparison. Then we utilize the principal component analysis (PCA) to obtain the slowest motions for each individual system from MD trajectories, which are further used to determine the reaction coordinate of conformational transition in the free energy calculations. Finally, we use metadynamics to compare the free energy profiles (FEPs) of conformational transitions and ligand binding process after replacing Ser35 by Pro in GK enzyme.

### The analysis of MD simulation trajectories

**Figure 2:**
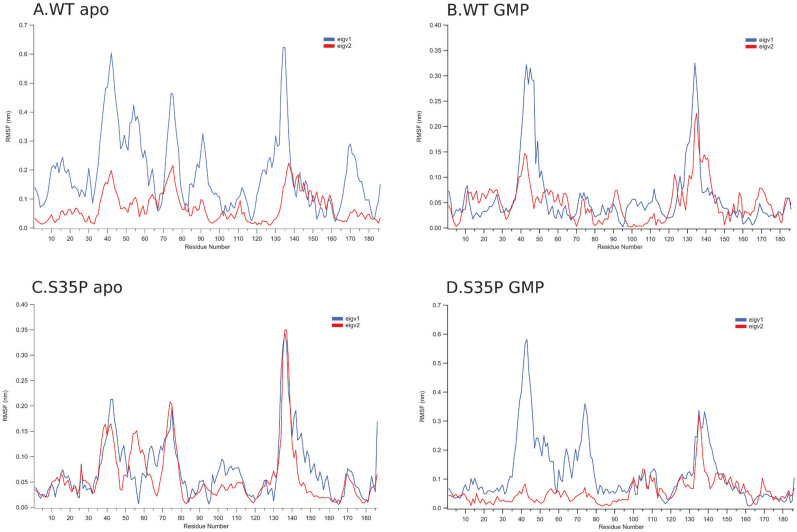
The root mean square fluctuations (RMSF) contributed by first two eigenvectors of the MD trajectories.

After performing the standard MD simulations of the five systems, we first analyzed each 300 ns MD trajectory using the principal component analysis. We find that it is sufficient to use the first two modes to describe the conformational changes of each system. In Figure 2, we depict the C-alpha RMSF contributed by the first two eigenvectors. As can be seen, the largest fluctuations during the transition between the closed and open conformations are observed in the hinge region between the beta3 and beta4 of the GBD domain (residues 40–46) with a peak at Gly43 and the loop region between the alpha5 and alpha6 of the LID domain (residues 135–141) with a peak at Thr137, respectively. Therefore, it is reasonable to define the distance between the Cα atoms of Gly43 and Thr137 as a collective variable (CV1) to describe the conformational changes between the GBD and LID domain of GK in our following metadynamics simulations. Note that the two positions determined by PCA were also used as labels to monitor the conformational transitions of GK in the fluorescence quenching the measurements performed by Johnston et.al^7^. Thus, our MD simulations can provide a direct comparison to the experimental data.

**Figure 3:**
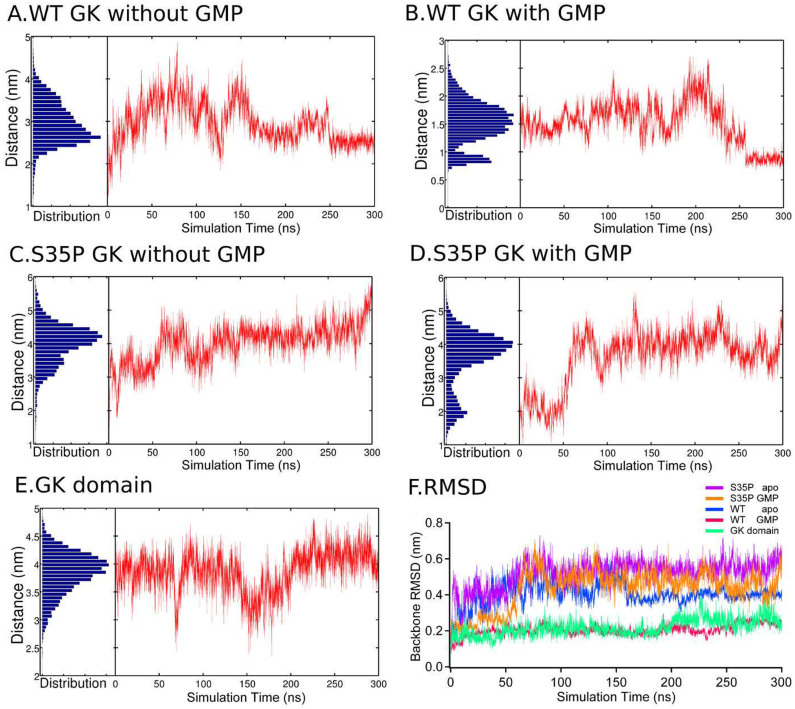
The distance profiles between the Cα atoms of Gly43 and Thr137 along the MD trajectory as well as the distributions. (A)the apo WT; (B)the GMP-bound WT; (C)the apo S35P mutant; (D) the GMP-bound S35P; (E)the GK domain and (F)the backbone RMSD.

In addition, we have analyzed the distance profiles between the Cα atoms of Gly43 and Thr137 as well as their distance distribution. The results are shown in Figure 3 and discussed in the following section.

### Dynamic behaviors obtained by standard MD simulations

#### The apo GK exhibits the partially closed conformation

**Figure 4:**
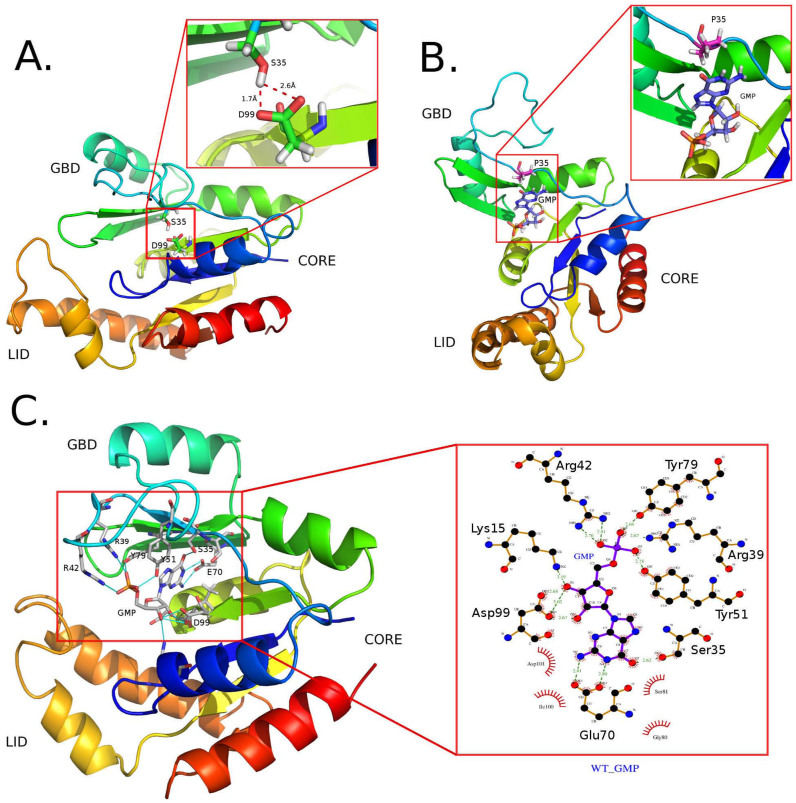
Snapshots of the stable conformations randomly selected from the last 50 ns MD trajectory. (A) The partially closed apo WT GK. The insert indicates the key interaction between Ser35 and Asp99 in maintain the partially closed conformation; (B) The open GMP-bound S35P GK. The insert indicates no hydrogen-bond formed between Pro35 and GMP; (C) the fully closed GMP-bound WT GK. The insert shows the hydrogen-bond interactions between GMP and the WT GK.

Since the initial conformation of the apo GK is prepared by removing the ligand from its GMP-bound state, we find that the apo GK enzyme experiences large fluctuations in the first 250 ns of our simulation. As displayed in Figure 3, the distance distribution between the GBD and LID domain of apo GK is the broadest compared to other systems, indicating a large sampled conformational space, which fluctuates from 1.5 nm to 4.5 nm. After 250 ns, the protein reaches a stable state with a distance of around 2.5 nm between the GBD and LID domain, corresponding to the formation of the partially closed conformation. After closely inspecting these structures, we find that a hydrogen bond, which is essential to maintain the partially closed conformation, is formed between Ser35 and Asp99, implicating a critical role of Ser35 (Figure 4).

### The GMP-bound GK maintains the fully closed state

**Figure 5:**
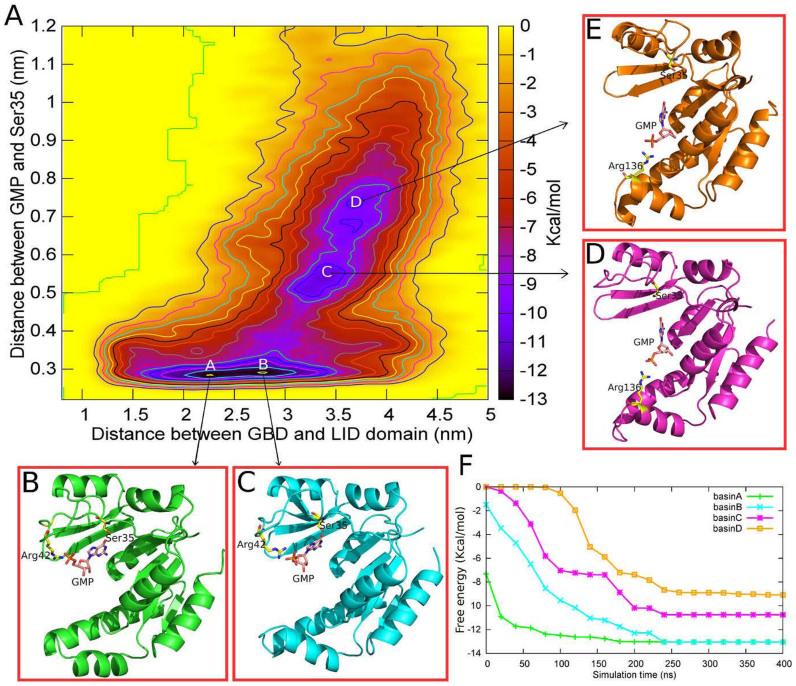
(A) The two-dimensional free energy profile (FEP) of the closed-open conformational transitions vs the GMP disassociation of WT GK. The separation between the contours is 1 kcal/mol. The four basins (A–D) represent the lowest four free energy minima of the FEP; (B–E)The conformations representative of the interactions between the residues and GMP of each basin; (F)The convergence of the four basins along the simulation. The free energy of each basin is converged after 300 ns.

In the presence of GMP, the inter-domain motions of GK enzyme are significantly restricted. The protein experiences only small fluctuations with backbone atoms RMSD around 0.2 nm during the whole simulation (Figure 5). The distribution of the distance between the GBD and LID domain also spans a small range from 0.8 nm to 2.5 nm (Figure 3). After 250 ns, the protein is stabilized in a fully closed state. Notably, the stable conformations obtained by our simulation share several key characteristics with the structures obtained by X-ray crystallography^6^,^7^,^8^,^9^ including: the phosphate group of GMP is stabilized by Tyr51, Tyr79, Arg39 and Arg42;the sugar hydroxyl group interacts with Lys15 and Asp99; the guanine carbonyl oxygen is hydrogen bonded by Ser35; the guanine amino group is interacted by Glu70. To have a better illustration of these interactions, we depict the detailed hydrogen-bond pattern in Figure 4. As can be seen from that figure, the presence of GMP interrupts the hydrogen-bond interaction between the Ser35 and Asp99 in the apo form and, in turn, the two residues change their roles in modulating the ligand specificity and affinity.

### Open conformations are more favorable in both apo and holo forms of S35P GK

Experimental data suggest that the Ser35→Pro mutation alters the GK function by changing its dynamic behavior. Therefore, in order to monitor the conformational transitions, we performed MD simulations of S35P GK by using the identical closed conformations in both ligand-free and ligand-bound forms (Figure 1). As expected, our simulations show that the dynamic behavior of the S35P GK is quite different from that of the WT GK. In the case of the GMP-bound S35P GK, we observe that the closed conformation is only maintained in the first 50 ns. However, due to the lack of interaction between the proline and the guanine carbonyl oxygen of GMP, the ligand is extremely unstable in the binding site. After 60 ns, the open conformations are achieved in both apo and holo forms of S35P GK with a large cleft between the GBD and LID domain. In the open state of holo form, the phosphate group of GMP may still interacts with the Arg39 and Arg42 located in the GBD domain for a long period of MD simulation time, which is in agreement with NMR observation that the GMP-induced chemical shift in the S35P GK could only be identified in the region of GBD domain^5^. During the rest of our simulations, the S35P mutant exhibits a high stability in the open conformation. At end of our simulation, we find that S35P GK loses the ability of ligand binding and the GMP escapes into the bulk solvent eventually.

### The GK domain demonstrates an open state like the S35P GK

As we mentioned above, mutating Ser35 to Pro in GK would intrinsically alter the dynamic behavior of the protein and lead to the open conformation more favorable regardless of the presence or absence of GMP. Then, we conduct an additional simulation of GK domain to identify whether the S35P mutant and the GK domain share similar conformational dynamics. Indeed, we find that the protein dynamics of the S35P GK and the GK domain are quite similar in their open conformations. A large cleft between the GBD and LID domain is maintained during the whole simulation in the GK domain, with the distance around 4.0 nm. In addition, the protein demonstrates a high stability with the backbone RMSD fluctuating only around 0.2 nm (Figure 3). All these dynamic features of GK domain are in line with its functional role that 1) the conformational rigidity of GK domain is essential to function as a module of scaffold protein, which provides the physical constraints for efficient signal transduction and 2) the stable open conformation is required to provide a large protein-binding surface for the binding of the peptide ligands.

Thus, our MD results can provide an explanation in support of the experimental evidence that the S → P mutation prevents the closing motions of GK and then allows the protein to function as a protein interaction domain^5^. Furthermore, the S → P mutation diminishes the GMP binding ability of GK Consequently, the enzymatic activity of the mutant is significantly impaired^6^.

### Reconstructing the free energy profiles using metadynamics

To further investigate the relationship between the conformational transition and the ligand binding properties in both WT and S35P GK, we employ metadynamics to reconstruct the two-dimensional free energy profiles (FEPs) along the reaction coordinates of CV1 and CV2. Our FEP results clearly reveal that, in the WT GK, the ligand binding process occurs concurrently with the conformational transitions, which is in line with the experimental observation that the closing motions of WT GK are induced by the binding of GMP7. As shown in Figure 5,the lowest free energy well is identified in a narrow region with CV1 ranging from 2.0 nm to 3.1 nm and CV2 less than 0.3 nm. In addition, the well can be further divided into two parts, corresponding to the fully closed (CV1 ≈ 2.2 nm) and the partially closed (CV1 ≈ 2.8 nm) states, separated by a small energy barrier less than 1 kcal/mol. The second lowest free energy well is identified in a broad region on the free energy surface ranging from 3.2 to 4.0 nm of CV1 and 0.48 to 0.80 nm of CV2, respectively, which corresponds to the process of conformational transitions between the partially closed to the open state and the ligand binding event. It is interesting to note that, as the conformation changes from the partially closed to the open state, there is a strong positive correlation between CV1 and CV2. This result provides a clear picture for the GMP-induced closing motions of WT GK.

After performing more analysis of the MD trajectories, we find that the second lowest minimum reflects the hydrogen-bond interactions between the phosphate group of GMP and the residue Arg136 located in the LID domain in the open conformations (Figure 5), suggesting the role of Arg136 in phosphate recognition. However, the FEP shows that the open state (basin D) is less energetically favorable than the closed state (basin A) in GMP binding with ΔG = ~4 kcal/mol (Figure 5).

Accordingly, our FEP calculations suggest that the binding process of GMP in WT GK occurs in two successive steps: 1) in the open state, the attraction of GMP is made by the interactions between the phosphate group of GMP and the Arg136 located in the LID domain which induces a trap for the ligand in the cleft between the GBD and LID domain; 2) the hydroxyl group of Ser35 tends to interact with the carbonyl oxygen of GMP via hydrogen-bond. Once the connection is formed between the two groups, the closing motion of GK occurs and the protein equilibrates between the partially closed and fully closed states. Meanwhile, the phosphate group of GMP translocates into a stronger binding site formed by Arg39 and Arg42 located in the GBD domain (Figure 5).

**Figure 6:**
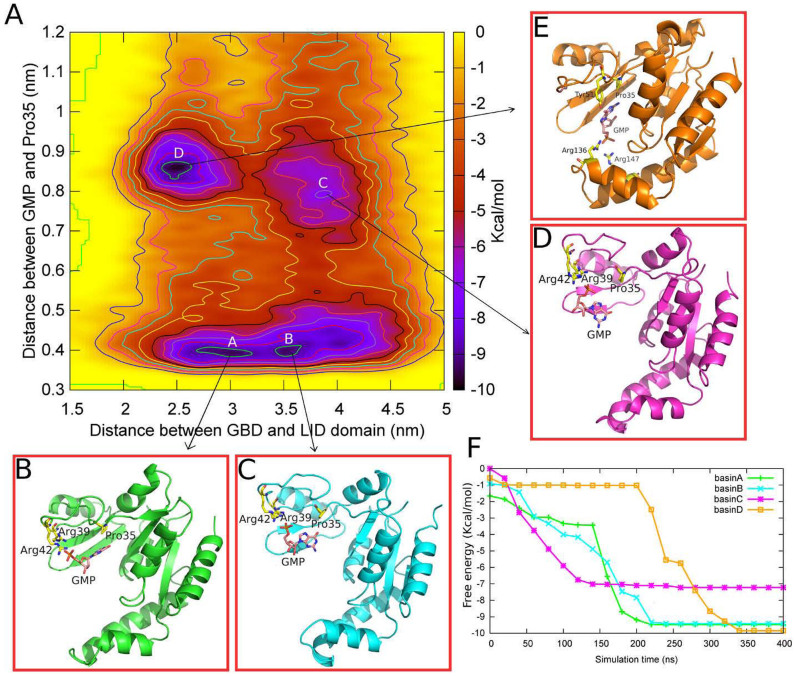
(A) The two-dimensional free energy profile (FEP) of the closed-open conformational transitions vs the GMP disassociation of S35P GK. The separation between the contours is 1 kcal/mol; The four basins (A–D) represent the lowest four free energy minima of the FEP; (B–E)The conformations representative of the interactions between the residues and GMP of each basin; (F)The convergence of the four basins along the simulation. The free energy of each basin is converged after 350 ns.

On the other hand, in the case of S35P GK, we find that the conformational transitions are not coupled with the process of GMP binding. As shown in Figure 6, the pathways of the two events are perpendicular to each other, indicating the two processes occur independently. In the conformational transition pathway, the protein undergoes no significant energy barriers (<1 kcal/mol) from 2.5 nm to 4.3 nm of CV1 even in the presence of GMP at the binding site (CV2 ≈ 0.4 nm). Compared with the WT GK, we observe that the lowest free energy well shifts as much as 10Å toward the open conformation in the S35P GK, suggesting that the closing motions of S35P GK are energetically unfavorable. The ligand dissociation from the binding site only occurs in the open state of S35P GK at CV1 ≈ 3.8 nm. After crossing an energy barrier of ~5 kcal/mol, the mutant reaches a metastable free energy minimum at CV1 = 3.8 nm and CV2 = 0.8 nm (Figure 6). In this open conformation, the phosphate group of GMP may still interact with two arginine residues Arg39 and Arg42 located in the GBD domain. This observation is in consistent with the results of our standard MD simulation and the experimental data^7^ mentioned above. In addition, the FEP shows that there is another deeper free energy well at CV1 ≈ 2.5 nm and CV2 ≈ 0.85 nm in the S35P GK, corresponding to the basin D in Figure 6. Tracing back to the MD trajectories, we find that this free energy minimum represents a mis-binding pattern in the closed conformation. In this state, the S35P GK is not able to recognize the ligand correctly because of the lack of the hydrogen-bond interaction between Pro35 and GMP. Instead, a hydrogen-bond is formed between the phenolic hydroxy group of Tyr51 and the guanine carbonyl oxygen of GMP, which results in a flip of the guanine group of GMP in the binding site. Furthermore, the binding of the phosphate group of GMP is also affected that the phosphate group binds to the two arginine residues Arg136 and Arg147 located in the LID domain instead of the arginines in the GBD domain. Taken together, the calculated FEPs further emphasize the critical role of Ser35 in ligand recognition and provide an explanation to the loss of the enzyme activity in the S35P mutant.

## Conclusion

In this work, we carried out molecular dynamic simulations to investigate the underlying mechanism of conformational transitions after replacing serine35 with proline in GK enzyme. Our MD simulations demonstrated that the single mutation intrinsically alters the dynamic behavior of the protein. In the apo GK, the Ser35 plays a key role in maintaining the partially closed conformation via the hydrogen-bond interactions with Asp99. In the presence of GMP, the Ser35 is critical for modulating the ligand specificity and affinity.

On the contrary, the S35P GK exhibits stable open conformations regardless of the presence or absence of GMP. The cleft between the GBD and the LID domain in S35P GK is as large as that exists in the GK domain, explaining the functional change of S35P GK to the GK domain.

To investigate the relationship between conformational transitions and ligand binding process in both WT and S35P GK, we further use metadynamics to calculate the free energy profiles along the two corresponding reaction coordinates. Our FEPs demonstrate that the closed-open conformational transition of WT GK is positively correlated with the GMP binding process, which strongly supports the experimental observation that the closing motion of GK is induced by the binding of GMP. In contrast, the S35P GK energetically favors the open conformation even with the presence of GMP. In addition, we also identify the mis-binding pattern in the S35P GK because of the lack of the hydrogen-bond interaction between proline 35 and GMP, implying the essential role of Ser35 in ligand recognition.

## Author Contributions

G.L. designed the idea. Y.Z., H.N. performed the MD simulations. All authors analyzed the data and prepared the figures. G.L. and Y.Z. wrote the manuscript with contributions from the other authors.

## References

[b1] Harms M. J. & Thornton J. W. Analyzing protein structure and function using ancestral gene reconstruction. Curr. Opin. Struct. Biol. 20, 360–366 (2010). 20413295 10.1016/j.sbi.2010.03.005 PMC2916957

[b2] Prince V. E. & Pickett F. B. Splitting pairs: the diverging fates of duplicated genes. Nat. Rev. Genet. 3, 827–837 (2002). 12415313 10.1038/nrg928

[b3] Konrad M. Cloning and expression of the essential gene for guanylate kinase from yeast. J. Biol. Chem. 267, 25652–25655 (1992). 1334480

[b4] Moriguchi M., Kohno H., Kamei M. & Tochikura T. Purification and properties of guanylate kinase from baker's yeast. Biochim. Biophys. Acta. 662, 165–167 (1981). 6118179 10.1016/0005-2744(81)90239-4

[b5] Oeschger M. P. Guanylate kinase from Escherichia coli B. Methods. Enzymol. 51, 473–482 (1978). 211389 10.1016/s0076-6879(78)51065-3

[b6] Stehle T. & Schulz G. E. Three-dimensional structure of the complex of guanylate kinase from yeast with its substrate GMP. J. Mol. Biol. 211, 249–254 (1990). 1967656 10.1016/0022-2836(90)90024-G

[b7] Johnston C. A., Whitney D. S., Volkman B. F., Doe C. Q. & Prehoda K. E. Conversion of the enzyme guanylate kinase into a mitotic-spindle orienting protein by a single mutation that inhibits GMP-induced closing. Proc. Natl. Acad. Sci. 108, E973–E978 (2011). 21990344 10.1073/pnas.1104365108 PMC3207680

[b8] Blaszczyk J., Li Y., Yan H. & Ji X. Crystal structure of unligated guanylate kinase from yeast reveals GMP-induced conformational changes. J. Mol. Biol. 307, 247–257 (2001). 11243817 10.1006/jmbi.2000.4427

[b9] Sekulic N., Shuvalova L., Spangenberg O., Konrad M. & Lavie A. Structural characterization of the closed conformation of mouse guanylate kinase. J. Biol. Chem. 277, 30236–30243 (2002). 12036965 10.1074/jbc.M204668200

[b10] Olsen O. & Bredt D. S. Functional analysis of the nucleotide binding domain of membrane-associated guanylate kinases. J. Biol. Chem. 278, 6873–6878 (2003). 12482754 10.1074/jbc.M210165200

[b11] Choi B. & Zocchi G. Guanylate kinase, induced fit, and the allosteric spring probe. Biophys. J. 92, 1651–1658 (2007). 17142284 10.1529/biophysj.106.092866 PMC1796832

[b12] Delalande O., Sacquin-Mora S. & Baaden M. Enzyme closure and nucleotide binding structurally lock guanylate kinase. Biophys. J. 101, 1440–1449 (2011). 21943425 10.1016/j.bpj.2011.07.048 PMC3177050

[b13] Velthuis A. J., Admiraal J. F. & Bagowski C. P. Molecular evolution of the MAGUK family in metazoan genomes. BMC Evol. Biol. 7, 129 (2007). 17678554 10.1186/1471-2148-7-129 PMC1978500

[b14] Mendoza A., Suga H. & Ruiz-Trillo I. Evolution of the MAGUK protein gene family in premetazoan lineages. BMC Evol. Biol. 10, 93–93 (2010). 20359327 10.1186/1471-2148-10-93 PMC2859873

[b15] Li Y., Spangenberg O., Paarmann I., Konrad M. & Lavie A. Structural basis for nucleotide-dependent regulation of membrane-associated guanylate kinase-like domains. J. Biol. Chem. 277, 4159–65 (2002). 11729206 10.1074/jbc.M110792200

[b16] Xu W. PSD-95-like membrane associated guanylate kinases (PSD-MAGUKs) and synaptic plasticity. Curr. Opin. Neurobiol. 21, 306–312 (2011). 21450454 10.1016/j.conb.2011.03.001 PMC3138136

[b17] Nomme J. *et al.* The Src homology 3 domain is required for junctional adhesion molecule binding to the third PDZ domain of the scaffolding protein ZO-1. J. Biol. Chem. 286, 43352–43360 (2011). 22030391 10.1074/jbc.M111.304089 PMC3234847

[b18] Funke L., Dakoji S. & Bredt D. S. Membrane-associated guanylate kinases regulate adhesion and plasticity at cell junctions. Annu. Rev. Biochem. 74, 219–45 (2005). 15952887 10.1146/annurev.biochem.74.082803.133339

[b19] Oliva C., Escobedo P., Astorga C., Molina C. & Sierralta J. Role of the MAGUK protein family in synapse formation and function. Dev. Neurobiol. 72, 57–72 (2012). 21739617 10.1002/dneu.20949

[b20] Mori S. *et al.* Crystal structure of the guanylate kinase domain from discs large homolog 1 (DLG1/SAP97). Biochem. Biophys. Res. Commun. 435, 334–338 (2013). 23624197 10.1016/j.bbrc.2013.04.056

[b21] Daniels D. L., Cohen A. R., Anderson J. M. & Brünger A. T. Crystal structure of the hCASK PDZ domain reveals the structural basis of class II PDZ domain target recognition. Nat. Struct. Biol. 5, 317–325 (1998). 9546224 10.1038/nsb0498-317

[b22] Zhu J. *et al.* Guanylate kinase domains of the MAGUK family scaffold proteins as specific phospho-protein-binding modules. EMBO J. 30, 4986–4997 (2011). 22117215 10.1038/emboj.2011.428 PMC3243629

[b23] Todorova N., Marinelli F., Piana S. & Yarovsky I. Exploring the folding free energy landscape of insulin using bias exchange metadynamics. J. Phys. Chem. B 113, 3556–3564 (2009). 19243106 10.1021/jp809776v

[b24] Humphrey W., Dalke A. & Schulten K. VMD: visual molecular dynamics. J. Mol. Graph. 14, 33–38 (1996). 8744570 10.1016/0263-7855(96)00018-5

[b25] Pronk S. *et al.* GROMACS 4.5: a high-throughput and highly parallel open source molecular simulation toolkit. Bioinformatics 29, 845–854 (2013). 23407358 10.1093/bioinformatics/btt055 PMC3605599

[b26] Van Der Spoel *et al.* GROMACS: fast, flexible, and free. J. Comput. Chem. 26, 1701–1718 (2005). 16211538 10.1002/jcc.20291

[b27] Duan Y. *et al.* A point-charge force field for molecular mechanics simulations of proteins based on condensed-phase quantum mechanical calculations. J. Comput. Chem. 24, 1999–2012 (2003). 14531054 10.1002/jcc.10349

[b28] Parrinello M. & Rahman A. Polymorphic transitions in single crystals: A new molecular dynamics method. J. Appl. Phys. 52, 7182–7190 (1981).

[b29] Berendsen H. J. C., Postma J. P. M., van Gunsteren W. F., DiNola A. & Haak J. R. Molecular dynamics with coupling to an external bath. J. Chem. Phys. 81, 3684–3690 (1984).

[b30] Essmann U. *et al.* A smooth particle mesh Ewald method. J. Chem. Phys. 103, 8577–8593 (1995).

[b31] Miyamoto S. & Kollman P. A. Settle: An analytical version of the SHAKE and RATTLE algorithm f rigid water models. J. Comp. Chem. 13, 952–962 (1992).

[b32] Hess B., Bekker H., Berendsen H. J. C. & Fraaije J. G. E. M. LINCS: A linear constraint solver for molecular simulations. J. Comp. Chem. 18, 1463–1472 (1997).

[b33] Leone V., Marinelli F., Carloni P. & Parrinello M. Targeting biomolecular flexibility with metadynamics. Curr. Opin. Struct. Biol 20, 148–154 (2010). 20171876 10.1016/j.sbi.2010.01.011

[b34] Branduardi D., Gervasio F. L. & Parrinello M. From A to B in free energy space. J. Chem. Phys. 126, 054103 (2007). 17302470 10.1063/1.2432340

[b35] Bussi G., Laio A. & Parrinello M. Equilibrium free energies from nonequilibrium metadynamics. Phys. Rev. Lett. 96, 090601 (2006). 16606249 10.1103/PhysRevLett.96.090601

[b36] Tiwary P. & Parrinello M. From metadynamics to dynamics. Phys. Rev. Lett. 111, 230602 (2013). 24476246 10.1103/PhysRevLett.111.230602

[b37] Barducci A., Bonomi M., Prakash M. K. & Parrinello M. Free-energy landscape of protein oligomerization from atomistic simulations. Proc. Natl. Acad. Sci. 110, E4708–E4713 (2013) 24248370 10.1073/pnas.1320077110 PMC3856838

[b38] Limongelli V., Bonomi M. & Parrinello M. Funnel metadynamics as accurate binding free-energy method. Proc. Natl. Acad. Sci. 110, 6358–6363 (2012). 23553839 10.1073/pnas.1303186110 PMC3631651

[b39] Todorova N., Marinelli F., Piana S. & Yarovsky I. Exploring the folding free energy landscape of insulin using bias exchange metadynamics. J. Phys. Chem. B 113, 3556–3564 (2009). 19243106 10.1021/jp809776v

[b40] Baftizadeh F., Cossio P., Pietrucci F. & Laio A. Protein Folding and Ligand-Enzyme Binding from Bias-Exchange Metadynamics Simulations. CPC 2, 79–91(2012).

[b41] Bonomi M. *et al.* PLUMED: A portable plugin for free-energy calculations with molecular dynamics. Comput. Phy. Commun. 180, 1961–1972 (2009).

[b42] Tribello G. A., Bonomi M., Branduardi D., Camilloni C. & Bussi G. PLUMED 2: New feathers for an old bird. Comp. Phys. Commun. 185, 604–613 (2014).

[b43] Barducci A., Bussi G. & Parrinello M. Well-tempered metadynamics: a smoothly converging and tunable free-energy method. Phys. Rev. Lett. 100, 020603 (2008). 18232845 10.1103/PhysRevLett.100.020603

[b44] Chen J., Zhang D., Zhang Y. & Li G. Computational Studies of Difference in Binding Modes of Peptide and Non-Peptide Inhibitors to MDM2/MDMX Based on Molecular Dynamics Simulations. Int. J. Mol. Sci. 13, 2176–2195 (2012). 22408446 10.3390/ijms13022176 PMC3292015

[b45] Shen H., Sun H. & Li G. What is the role of motif D in the nucleotide incorporation catalyzed by the RNA-dependent RNA polymerase from poliovirus? PLoS Comput. Biol. 8, e1002851 (2012). 23300428 10.1371/journal.pcbi.1002851 PMC3531290

[b46] Sun H., Chu H., Fu T., Shen H. & Li G. Theoretical Elucidation of the Origin for Assembly of the DAP12 Dimer with Only One NKG2C in the Lipid Membrane. J. Phys. Chem. B 117, 4789–4797 (2013). 23560746 10.1021/jp312375g

[b47] Zhang Y., Shen H., Zhang M. & Li G. Exploring the proton conductance and drug resistance of BM2 channel through molecular dynamics simulations and free energy calculations at different pH conditions. J. Phys. Chem. B 117, 982–988 (2013). 23286443 10.1021/jp309682t

